# Perspectives from individuals with low education and interviewers using the GloboDiet 24 h recall: a qualitative study

**DOI:** 10.1017/jns.2020.6

**Published:** 2020-04-01

**Authors:** Nathalie A. S. Koubik, Caroline O. Medeiros, Glenda V. da Silva, Juliana B. Gonçalves, Sandra P. Crispim

**Affiliations:** 1 Postgraduate Program in Food and Nutrition, Department of Nutrition, Federal University of Paraná (UFPR), Curitiba 80240-110, Brazil; 2 Department of Nutrition, Federal University of Paraná (UFPR), Curitiba 80240-110, Brazil; 3 Faculdade Paranaense (FAPAR), Curitiba 80420-060, Brazil

**Keywords:** Food intake, Diet, Interviews, Education, Focus groups, R24h, 24-h recall

## Abstract

The perception of individuals with low education about dietary assessments is not well explored and studying this may be beneficial to improve data collection. The study builds on previous quantitative studies by providing explanations for the observed lower performance of the 24-h recall method among low-educated individuals. A qualitative study was carried out in Brazil. First, trained interviewers attended a focus group via video conference. Next, individuals with low education, defined as less than 9 years of study, participated in semi-structured face-to-face interviews. Three main themes emerged from the focus groups and were contrasted with the interviews. Summarising, the establishment of adequate communication during the interview is of utmost importance among the low-educated population. Besides, the familiarity of individuals with food and nutrition favours the report of information. Lastly, the use of photographs for food portion quantification helps the dietary assessment although further investigations to improve their use are also needed.

Dietary assessment is a valuable source of information for action planning in the food and nutrition field. However, this is a complex task, as food intake may be under- or overestimated, resulting in the production of data that are not compatible with reality^(1)^. Therefore, technological innovations, such as the development of computer-based 24-h recalls (R24h), have been proposed for data collection in epidemiological and diet-monitoring studies^(2–4)^, aiming to reduce biases and to enhance the methods for dietary assessment^(5)^.

As an example, the computer and interview-based R24h GloboDiet was developed by the International Agency for Research on Cancer (IARC) to standardise food intake data of the adult population worldwide, including Latin America^(3,6)^. In Brazil, an adapted version of the software together with a photograph manual for food portion sizes is available^(7)^. Nonetheless, when we assessed the ability of individuals to quantify some of those photographs, we noticed larger errors among low-educated individuals compared with other population groups^(8)^. Although individuals are cognoscenti^(9)^ and education level is usually considered not to be a limiting factor for the R24h^(10)^, the level of education seemed to influence the ability of those interviewed to inform their food intake.

Additionally, despite new technologies being considered good initiatives for the collection of dietary intake^(11)^, the investigation of their use and validity in different population groups is essential. Likewise, the assessment of qualitative aspects to clarify the reasons why the performance of the methods is distinguished among different groups is useful, especially in low- to middle-income countries. Since low education is a characteristic still present in many contexts, studying the perception of such individuals as well as analysing the perceptions of the interviewers responsible for the application of dietary interviews, including R24h, may improve the strategies on the collection of dietary intake data. In Brazil, for instance, more than half of its population aged 25 years or more are not completing the compulsory basic education^(12)^.

Therefore, our study aimed to assess the perception of individuals with low education and their interviewers when performing a R24h interview using the GloboDiet software.

## Methods

The present research is an exploratory qualitative study developed at the Federal University of Paraná (UFPR), in Brazil. The study comprised a focus group including interviewers responsible for the GloboDiet software application in Brazil complemented with semi-structured interviews carried out in individuals with low education, who had previously answered a R24h GloboDiet interview in the university. Ethical clearance was obtained from the Research Ethics Committee in the Health Sciences Division (CEP/SD) of the UFPR, under the number 363.816, according to the Normative Ordinance no. 466/2012 of the National Health Council (CNS). All of the participants provided their informed consent.

### Recruitment of interviewers

Trained interviewers with experience in the application of the GloboDiet in Brazil were recruited. During the selection, there were three Brazilian research centres located in the South (Curitiba), Northeast (Sergipe) and Southeast (São Paulo) of the country, with dietitians and nutrition students trained to collect data using the software. The interviewers were first contacted through email. Information about the study was sent and a questionnaire was filled out, including questions about their identity and their experience with R24h recalls based on paper-and-pencil and/or computer as well as their knowledge regarding the so-called multiple-pass method, which is used to give structure to the interview on GloboDiet.

Out of the twelve interviewers who were contacted, nine of them showed interest. Two of them participated in a first testing focus group, one of them participated in a second testing, and the others (two from each centre) participated in the official focus group carried out in September 2017. Besides, the testing of focus groups included other participants who did not have experience in using the software.

The interviewers had different graduation levels at the time the data were collected: two of them were undergraduates in nutrition, and four of them were dietitians. Ages varied between 20 and 34 years, with a median of 25 years. All interviewers said they already knew the computer-based and the paper-and-paper approach of R24h, and also reported that they had applied both of them. All interviewers stated they knew and had applied the multiple–pass method for R24h. On average, the interviewers had 6 months of experience in using the GloboDiet software, ranging from 3 to 18 months.

### The focus group

**Fig. 1.**
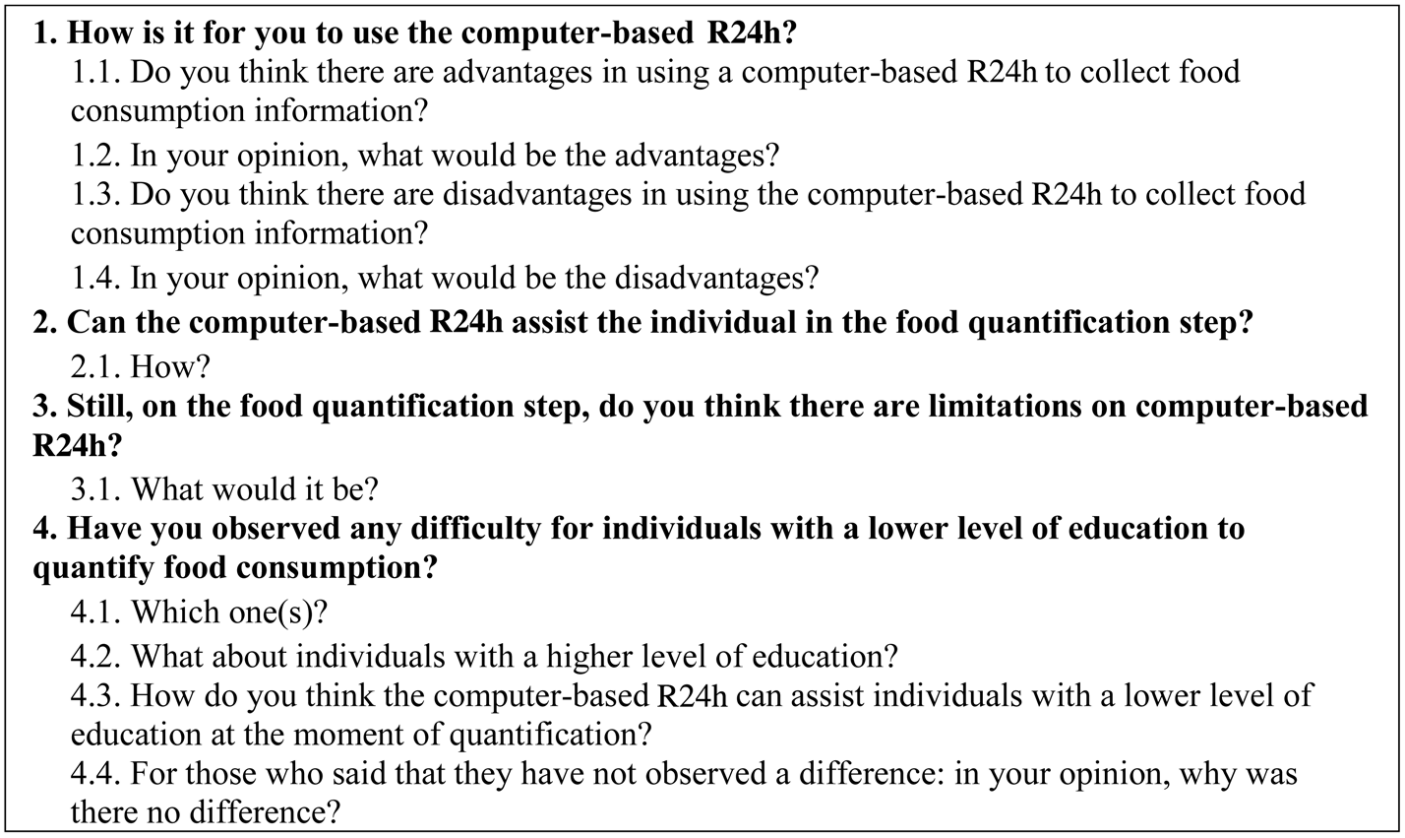
Guiding questions and sub-questions of the focus group with interviewers. R24h, 24-h recall

Based on both testing focus groups, guiding questions and sub-questions were improved before the official focus group (Fig. 1). As the interviewers were from different places in Brazil, the focus group meeting happened through video conference, which required all the participants to be online at the same time^(13)^. During the video conference, sounds and images were projected to all access points. Audio and video of the focus group were recorded. In Curitiba, the interviewers and researchers shared the video conference space. For that, they had to be placed in a position where they could see the screen and the other interviewers from the other centres. In São Paulo and Aracaju, the rooms were organised on the day of the conference by the participants, ensuring an adequate view of the screen and good visual contact with the other participants.

The focus group was moderated by one researcher and included two other observers. The moderator was responsible for leading the focus group, one observer took notes on the statements of the participants, and the other observer took notes on non-verbal communication as well as any possible complication.

### Recruitment of individuals with low education

Low-educated individuals with up to 9 years of study, and who had been interviewed with the GloboDiet R24h in another study in Curitiba, were invited to participate in a new interview. These were subjects participating in a different study. Thus, on the same day and after their R24h data collection, the selected participants answered a face-to-face semi-structured interview.

Two men and five women were interviewed, whose ages varied between 33 and 52 years (median of 48 years). They had a median of 5 years of study, ranging from 2 to 8 years. Subjects’ occupations included: housemaid, babysitter, janitor, maintainer and driver. Four of the respondents reported to be outsourced employees and three were only visiting the university.

### The semi-structured interviews

**Fig. 2.**
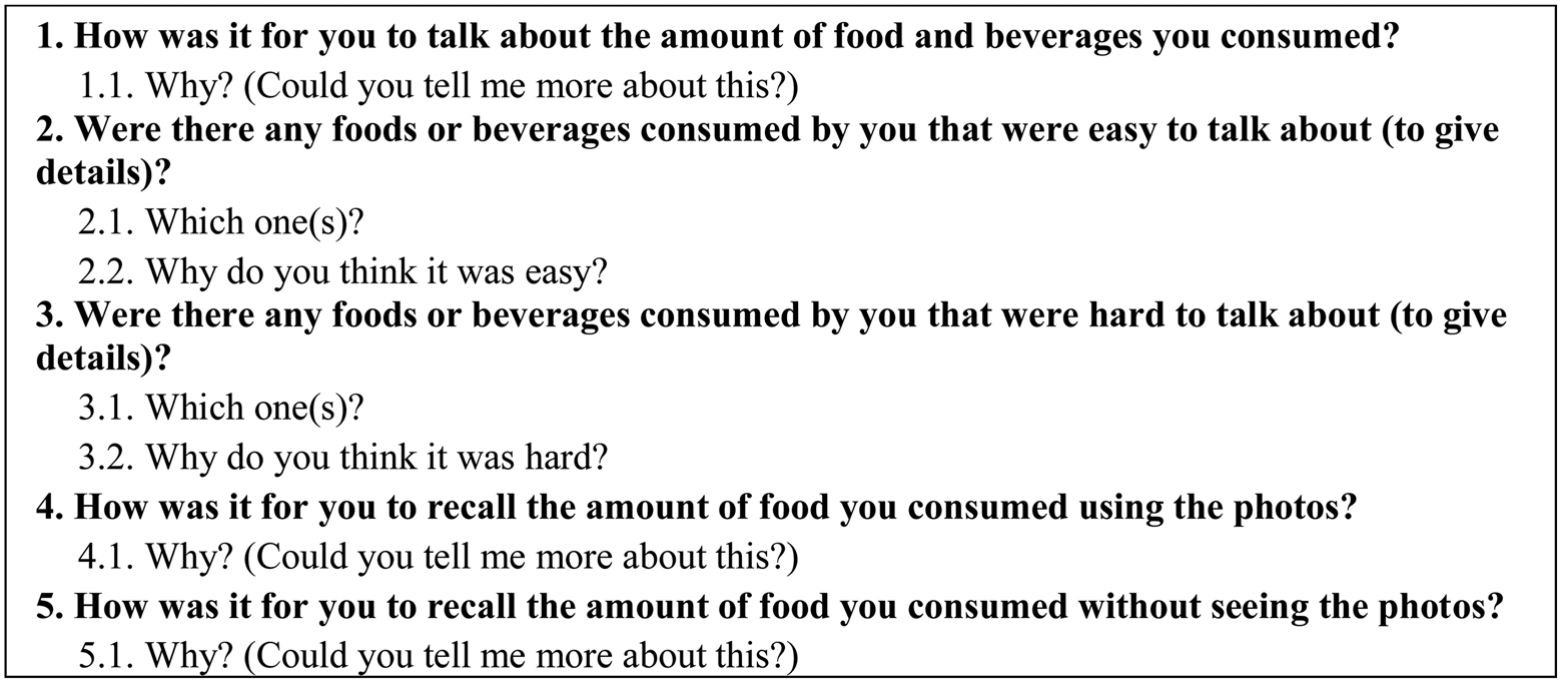
Guiding questions and sub-questions of the semi-structured interviews.

All individuals interviewed could talk freely about the topic proposed, without a correct answer or conditions imposed by the interviewers. The face-to-face interviews happened from November 2017 to April 2018, and they were carried out according to pre-defined questions and sub-questions (Fig. 2). The interviews were recorded with prior consent from the respondents. The semi-structured interviews took place in a room located in the university.

### Data analysis

First, the content of both focus group and interviews recorded was fully transcribed. After that, all data were analysed through the content analysis proposed by Bardin^(14)^, using a theme analysis following three steps proposed by the authors: (1) pre-analysis, (2) material study and (3) analysis, inference and interpretation of the results.

During the pre-analysis step, we carried out the operationalisation and systematisation of initial ideas based on exploratory data analysis. We also established operational goals, which were based on issues proposed for both the focus group and the semi-structured interviews, aiming to support the establishment of analysis categories and justify our final interpretation.

The analysis of the material comprised the organisation of the data considering three main points: (1) the recording units, which correspond to the segment of the content; (2) the context units, which can be understood as the comprehension units necessary for coding the registry units; (3) the themes, which represent the meaning units after naturally releasing a text that was analysed^(14)^. To have depth and complementarity in the discussion of the results, the categories from the focus group and the semi-structured interviews were considered simultaneously.

In the last analysis step, the meaning of the material, as well as its interpretation, were elaborated. It is important to highlight that unlike the semi-structured interview, the material from the focus group was considered to reflect the perceptions of the group and not of a specific individual^(15)^.

## Results

All interviewers participated actively during the discussion of the focus group, which lasted 73 min. In this discussion, we could notice agreements and disagreements through non-verbal communication among the interviewers, which were considered in the interpretation of the results. The total duration of the individual interviews was 18 min and 35 s. All interviewees were very cooperative.

**Fig. 3.**
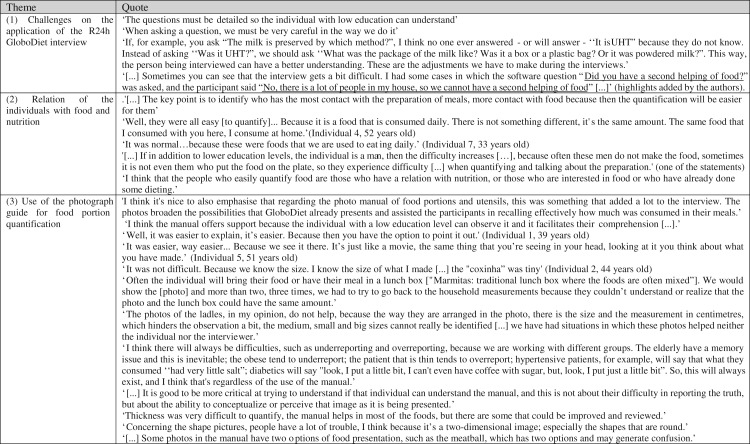
Emerging themes and sample quotes relating to the focus groups and interviews. R24h, 24-h recall

The theme analysis of the focus group and the semi-structured interviews resulted in three categories (Fig. 3). These were: (1) challenges on the application of the R24h GloboDiet interview; (2) relation of the individuals with food and nutrition; and (3) use of the photograph manual for food portion quantification.

### Challenges in the application of the 24-h recall GloboDiet interview

Among the challenges derived from the perceptions of interviewers and interviewees, stood out some problems to understand questions and to establish adequate communication as well as the importance of individual context regarding food access.

According to all interviewers, problems with understanding were very noticeable among individuals with a lower education level. This was due, especially, to the way the questions were made. All interviewers agreed that they needed to pay attention during the interview to make the necessary adjustments to the questions to better address these individuals.

The context regarding food access of low-educated individuals seems also to influence the R24h interview. The interview carried out with socio-economically vulnerable groups may be hampered due to discomfort to report what they eat, as it may expose their socio-economic situation and make them feel uncomfortable.

### Relationship of individuals with food and nutrition

In this category, we highlight a few aspects of the relationship of individuals with food and nutrition, which seems to improve the dietary assessment. In particular, all interviewers agreed that familiarity with food and its preparations and the interest of individuals in food and nutrition will influence the ability of low-educated individuals in reporting what they eat. In particular, five interviewers pointed out that the lack of familiarity interferes negatively in the moment of quantification. Some interviewers suggested that men with lower education levels, who have little contact with the preparation of their food, have more difficulty in detailing information. Furthermore, the interviewers stated that individuals who have been dieting in the past and/or were generally interested in food or nutrition knowledge appeared to quantify foods more easily.

### Use of the photograph manual for food portion quantification

The photographs of food portions used during the R24h seem to improve the interview with GloboDiet, as they allow the interviews to be faster. This was confirmed by all interviewers. Moreover, two interviewers noticed that the photographs provided greater agility in the interview of individuals with lower education levels, and facilitated the quantification step during their experience. As for the perception of those interviewed, they all stated that it was ‘easy’ to quantify the foods they had consumed when using the photographs.

However, some difficulties were observed by the interviewers during the use of the photographs by individuals with lower education levels, as well as in the use and analysis of the photographs. Three interviewers pointed out problems in the use of the photographs to quantify thickness and food shapes. Two interviewers also identified the lack of comprehension in the analysis of the photographs. However, it was also stressed by the interviewers that dietary measurement involves a subjective assessment and it will never be free from errors, regardless of any methodology or instrument used.

## Discussion

In the present study, interviewers and individuals with low education discussed their perception when performing a R24h interview using the GloboDiet software. This research builds on previous quantitative studies by providing context and explanations for the observed lower performance of the method among low-educated individuals.

The observed challenges in the application of the R24h interview in individuals with low education call attention to the need for changing the way of questioning food consumption to improve communication. Popular terminologies seem to be favoured over technical ones, as it can reduce comprehension problems and enhance the achievement of more reliable results. Individuals that live in contexts of social inequality, such as low education, may be unable to access the meaning of linguistic terms for a good understanding of the question and, therefore, give a good answer^(9)^. In addition, we need to understand how the person interviewed understands the question. By understanding this, the interviewer might learn about implied problems in his/her questions and try to fix them^(16)^.

Although problems are not always understood and their respective corrections made, we expect the interviewer to confirm if the interviewee is ready to carry out the interview^(17)^. Furthermore, supporting questions might minimise problems faced by those interviewed and favour the obtaining of more reliable statements^(18)^. However, even though a good communication is established with the interviewees and it shows positive results during the R24h interview, the interviewer must be careful when trying to help to not induce the answers, which would also cause errors^(10)^. This is not a simple task but we suggest that the investment in training is fundamental so interviewers can get acquainted with the instrument and the targeted group, especially the low-educated one.

Another important aspect, which will require attention and questioning adjustments by the interviewer, relates to the context of food access the interviewees have. Some of the observed statements may reveal the desire of the individuals to have a better diet or even their prejudgment about what they consume, coupled with the fear of possible criticism from the interviewer. Thus, it is important to understand the context in which the individual is inserted^(19)^ and that the responsibility of the interviewer is not only to extract information for answering technical–scientific questions but also to consider that the individuals are exposed while trying to answer them.

Furthermore, the level of knowledge about food and nutrition can influence the ability of individuals in quantifying food consumption. It is suggested that the fact that individuals have little contact with food and nutrition can lead to the production of answers considered to be less satisfactory. This may reflect the context of the individual, and do not necessarily represent a difficulty or have a relationship with the level of education. As a practical suggestion, it seems to be important to get to know those who are being interviewed and understand the limitations and potentialities that emerge from the individuals’ characteristics to allow interviewers to better guide the dietary interview.

As an important example, male individuals with a lower education level, who do not have contact with food preparation, appear to have more difficulties in quantifying food consumption. This result may be related to the fact that the presence of women still seems to be more representative in kitchen spaces and that women are still identified as great holders of knowledge about food^(20)^. We suggest, therefore, that the quality of food consumption quantification depends on the proximity that individuals have with food and this can be independent of sex, as long as both women and men are acquainted with food knowledge.

Regarding the use of the photographs for food portion estimations, quantitative studies usually suggest benefits in the use of photographs or lower performance in groups of lower education levels when compared with those of higher education levels^(21)^. In this perspective, studies have been and are being developed to improve the R24h method, including the use of additional visual aid resources, such as photographs, to reduce errors in diet measurement^(2,6)^.

In Brazil, the manual of photographs for food portion quantification was adapted to the reality of the Brazilian population. Even though the manual has been adapted and its use meets a significant demand for improving the quality and accuracy of quantitative data collected with 24 h recalls, the present results suggest that it is still necessary to have a deeper understanding of the difficulties and limitations encountered when using the photographs. In particular, further research is suggested about the use of the photographs in different population groups, along with investment in training and acquaintance of the interviewers with the targeted population.

Moreover, to minimise the difficulties encountered when using the photograph manual, the interviewers are encouraged to take the following measures: to study the photographs before conducting the interviews; adopt a standard use of the photographs when possible; allow those being interviewed to choose the photographs that help them the most, which may include household measurements, food portions, standard units or food shapes.

As a major strength of the study, we highlight that this is the first qualitative study to examine the perception of individuals about the assessment of diet in the low-education population and a low- to middle-income country. Among the limitations, the development of a single focus group may have produced only part of the considerations and reflections of the interviewers, and therefore limited the collection of new findings that would explain the perceptions in-depth. Furthermore, the interviewers did not have much experience with the software, as compared with professionals from other countries. However, they were the only trained interviewers at the time of the study in the country, limiting the inclusion of more participants. In addition, it could be questioned whether the focus group done via video conference presented different results from what would have been presented if the discussions were face-to-face. In fact, the use of video conferencing for focus groups has been an option given for performing qualitative research^(13)^. With that in mind, we tried to assure that the structure of the video conference focus group would be as similar as possible to the face-to-face one. There were no difficulties, all equipment worked continuously during the discussion and everyone could see and hear each other, with the addition of voice and video recording for checks. The moderator was also trained to conduct the conversation keeping up with the interaction among the different centres. As the most positive aspect of using video conferencing, we point out that most probably the reality of three different regions of Brazil would not have been captured otherwise. Lastly, the low education level of those interviewed in this study is likely to differ from the level of education of the population from other regions within Brazil and between other countries. We highlight, however, that the interviews were face-to-face and due to logistic constrictions, we did not interview individuals from other regions. Therefore, the results could have been different, which leaves room for further research on the subject.

### Conclusion

There are challenges to be considered when collecting information from individuals with low education, especially to make them understand what is being asked. It is important to mention that some aspects of the relationship of individuals with food and nutrition, such as familiarity, apparently favour the performance of the subjects when reporting food amounts. The use of the photograph manual during the R24h interview seemed to have provided improved dietary information collected with the software. However, interviewers also expressed concerns about the use of some pictures from the manual.

## Notes

Financial support was granted by the following Brazilian Agencies: Conselho Nacional de Desenvolvimento Científico e Tecnológico (CNPq; grant no. 441981/2014-3) and Coordenação de Aperfeiçoamento de Pessoal de Nível Superior – Brasil (CAPES; finance code 001).

N. A. S. K., C. O. M., J. B. G. and S. P. C. designed the study. N. A. S. K. and G. V. da S. were responsible for the data collection and analysis. N. A. S. K. and S. P. C. wrote the main draft of the manuscript. All authors contributed and reviewed the manuscript.
